# Association of chronic kidney disease (CKD) and failure to monitor renal function with adverse outcomes in people with diabetes: a primary care cohort study

**DOI:** 10.1186/1471-2369-14-198

**Published:** 2013-09-18

**Authors:** Andrew P McGovern, Benjamin Rusholme, Simon Jones, Jeremy N van Vlymen, Harshana Liyanage, Hugh Gallagher, Charles RV Tomson, Kamlesh Khunti, Kevin Harris, Simon de Lusignan

**Affiliations:** 1 Department of Healthcare Management and Policy, University of Surrey, Guildford, UK; 2 South West Thames Renal and Transplantation Unit, St Helier Hospital, Carshalton, UK; 3 Southmead Hospital, North Bristol NHS Trust, Bristol, UK; 4 John Walls Renal Unit, University Hospitals of Leicester NHS Trust, Leicester General Hospital, Leicester, UK

**Keywords:** Unmonitored, Primary care, Cardiovascular risk, Proteinuria, Estimated glomerular filtration rate

## Abstract

**Background:**

Chronic kidney disease (CKD) is a known risk factor for cardiovascular events and all-cause mortality. We investigate the relationship between CKD stage, proteinuria, hypertension and these adverse outcomes in the people with diabetes. We also study the outcomes of people who did not have monitoring of renal function.

**Methods:**

A cohort of people with type 1 and 2 diabetes (N = 35,502) from the Quality Improvement in Chronic Kidney Disease (QICKD) cluster randomised trial was followed up over 2.5 years. A composite of all-cause mortality, cardiovascular events, and end stage renal failure comprised the outcome measure. A multilevel logistic regression model was used to determine correlates with this outcome. Known cardiovascular and renal risk factors were adjusted for.

**Results:**

Proteinuria and reduced estimated glomerular filtration rate (eGFR) were independently associated with adverse outcomes in people with diabetes. People with an eGFR <60 ml/min, proteinuria, and hypertension have the greatest odds ratio (OR) of adverse outcome; 1.58 (95% CI 1.36-1.83). Renal function was not monitored in 4460 (12.6%) people. Unmonitored renal function was associated with adverse events; OR 1.35 (95% CI 1.13-1.63) in people with hypertension and OR 1.32 (95% CI 1.07-1.64) in those without.

**Conclusions:**

Proteinuria, eGFR < 60 ml/min, and failure to monitor renal function are associated with cardiovascular and renal events and mortality in people with diabetes.

## Background

Diabetes mellitus is an increasingly common condition and is associated with increased risk of cardiovascular events and mortality [1,2]. Furthermore, diabetes is the most common cause of end-stage renal disease (ESRD); in the United States it account for up to 45% of all new cases of ESRD [3]. Chronic kidney disease (CKD) is a risk factor for cardiovascular disease and mortality both in the general population [4,5] and amongst people with diabetes [6]. The two components of CKD; reduced estimated glomerular filtration rate (eGFR) and proteinuria have been shown to be independently associated with cardiovascular events and mortality in the general population and in high risk populations [7-9].

The National Institute for Health and Clinical Excellence (NICE) currently recommends all people with diabetes in England and Wales regularly have their renal function tested, including testing for albuminuria [10]. However, people with CKD complicating diabetes are not always identified and are sometimes sub-optimally managed in primary care [11]. Early identification of CKD and intervention with renoprotective measures, particularly the use of angiotensin converting enzyme inhibitors (ACEIs) and angiotensin receptor blockers (ARBs), has been shown to be effective in slowing progression of renal disease and in reducing cardiovascular events [12-16], and treatment that reduces proteinuria also reduces the risk of progression [17].

We investigated the association of eGFR and proteinuria on adverse vascular and renal outcomes in people with diabetes, with or without hypertension, in a community setting. In addition we investigated the association of these outcomes in people who did not have monitoring of their renal function.

## Methods

We performed a cohort analysis on all adults with diabetes in the Quality Improvement in Chronic Kidney Disease (QICKD) trial database. The QICKD trial was a three-armed cluster randomised controlled trial to analyse the impact of quality improvement interventions on blood pressure in people with renal disease [18]. Randomisation occurred at the primary care practice level. Practices were allocated to usual practice (no intervention), provision of clinical guidelines and prompts, or audit-based education. A reduction was of 2.41 mmHg (CI 0.59-4.29 mmHg; p = 0.012) was demonstrated with audit based education. However, neither intervention was found to have any impact on cardiovascular outcomes during the follow-up period. The trial database comprises routinely collected general practice (GP) data from 127 primary care practices across England; a nationally representative sample of urban, sub-urban and rural practices in London, Surrey, Sussex, Leicester, Birmingham and Cambridge between January 2006 and December 2010 [19]. There were additional records, of varying durations, for each person prior to these dates. Data recorded between January 2006 and June 2008 were used to determine the baseline characteristics of the people included in the study. A second data collection was undertaken at 30 months to obtain follow-up data.

We included all adults with type 1 and 2 diabetes. These patients were identified using a validated method for identifying correctly coded cases of diabetes from UK primary care records [20]. This method included analysis of diabetes read codes (e.g. read codes C10E and C10F are used for type 1 and type 2 diabetes respectively in UK primary care) in combination with documented investigation results. A person was defined as having diabetes if they had read codes for either type 1 or type 2 diabetes or laboratory values consistent with the World Health Organisation (WHO) diagnostic criteria for diabetes [21,22] prior to, or during the baseline period; a fasting blood glucose > 7.1 mmol/l, non-fasting blood glucose > 11.1 mmol/l, or HbA1c > 59 mmol/mol (7.5%). People with a recorded diagnosis of gestational diabetes, or elevated blood glucose results during pregnancy were excluded unless they subsequently developed diabetes outside of pregnancy.

England has a registration based primary health care system. With very few exceptions the whole population is registered with a single GP. Patients access non-emergency services through their GP and the GP receives letters about all hospital attendances including emergencies. Patients all have a unique ID called NHS number, which is attached to all their medical records. The health system has been progressively computerised since the 1990s [23]. Since 2004 there have been pay-for-performance indicators for chronic disease management, to which CKD was added in 2006 [23]. Remuneration for these is based on extracts from routine computer data [24]. Routinely collected primary care data in the UK, therefore, provides a highly comprehensive patient record.

### Outcomes

A composite outcome measure of incident stroke, transient ischaemic attacks (TIA), myocardial infarction (MI), death, advanced coronary artery disease, heart failure, and progression to end stage renal failure during the follow up period was used. Advanced coronary artery disease comprised revascularisation procedures (percutaneous coronary angioplasty and coronary bypass surgery) and patients with preinfarction syndrome. Preinfarction syndrome consists of newly recorded diagnosis of unstable angina, angina at rest, refractory angina, progressive angina, and acute coronary syndrome not otherwise recorded as a myocardial infarction.

### Predictors

Established predictors of adverse vascular and renal events were controlled for. These comprise: age, sex, ethnicity, deprivation status, smoking status, alcohol intake, body mass index (BMI); comorbid heart failure, hypertension, ischemic heart disease (IHD), advanced coronary artery disease, renal failure requiring dialysis, and dyslipidaemia; a previous history of stroke, transient ischemic attack (TIA), myocardial infarction (MI), coronary artery revascularisation procedures; and current use of aspirin, lipid lowering medication, ACEI or ARB medication, and other anti-hypertensive medications.

Demographic factors were extracted from GP records. Deprivation scores were derived from national statistics using patient postcodes at the point of data extraction (in compliance with data governance standards) [25]. Deprivation scores provide a combined measure of household income, education, healthcare provision, and living environment for the UK at small spatial scales [25]. Each locality is ranked by decile from most deprived to least deprived.

Smoking status and alcohol intake were based on the most recently recorded value for each factor before the start of the follow up period. Hypertension was defined as a recorded diagnosis of hypertension, or repeated blood pressure measurements greater than 130/80 mmHg [26-28]. A previous history of stroke, TIA, MI, or coronary artery revascularisation procedure and comorbid diagnoses of coronary heart failure, IHD, and dialysis were recorded as either present or absent based on the presence of a recorded diagnostic codes. Dyslipidaemia was subdivided into total cholesterol (TC), high density lipoprotein (HDL) cholesterol, and low density lipoprotein (LDL) cholesterol for analysis. Prescription records were used to define medication use. The additional risk factors analysed were: CKD stage, proteinuria (see below), urine albumin creatinine ratio (ACR), and most recent glycated haemoglobin (HbA_1C_) result.

The severity of CKD was stratified by stage, using the NICE definition [10], based on two eGFR measurements where available and the presence or absence of proteinuria. People with an eGFR of 60–90 ml/min or eGFR > 90 ml/min with proteinuria were classified as having CKD stages 1–2. People with an eGFR of < 60 ml/min were classified as having CKD stages 3–5. People who were on dialysis were also included in this category regardless of their creatinine measurements. eGFR values were calculated from serum creatinine using the 4-variable Modified Diet in Renal Disease (MDRD) equation [29,30]. This remains the standard equation used in UK primary care since the implementation of a national quality standard in 2006 for calculating eGFR across all laboratories in the UK.

**Figure 1:**
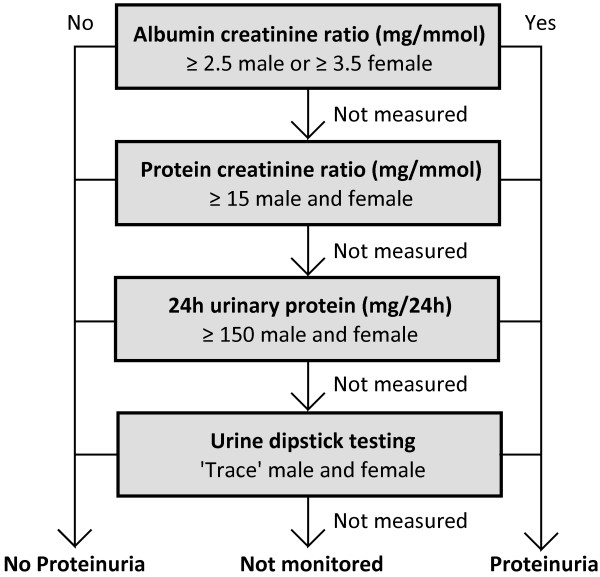
**Hierarchy of clinical tests used for the diagnosis of proteinuria.** Threshold values are adapted from the 2008 NICE guidelines [10] and Lamb et al. [31].

Proteinuria was analysed using the diagnostic criteria described by NICE [30]: The presence of proteinuria was determined by examining a hierarchy of clinical tests, with albumin creatinine ratio (ACR) being the highest (Figure 1). Where ACR testing had been done it was distinctly categorised by severity; normal (<2.5 mg/mmol males, <3.5 mg/mmol females), microalbuminuria (2.5–30 mg/mmol males, 3.5–30 mg/mmol females) and macroalbuminuria (>30 mg/mmol) [31].

People were defined as having unmonitored renal function if they did not have an eGFR recording and urine protein test during the 30 month baseline period.

### Statistical analysis

The data were first refined, to adjust for inputting errors, by removing numeric values above or below the maximum physiological limits. A complete case analysis was performed to account for the effects of loss to follow-up. A multilevel binary logistic regression model was built to account for variation between primary care practices. Patients were nested within GP practice using a random intercept. This was performed using the statistical package R and the multilevel R package lme4 [32]. Model selection was performed using the approach described by Maindonald and Braun [33]; minimising the Bayesian information criterion using backward stepwise elimination. Time to event models (Cox proportional hazard models) were also produced using the statistical package R. Model validation was performed for all models.

A Kaplan-Meier survival analysis was conducted to identify event free survival differences by CKD stage in people with and without hypertension. A logrank test was performed.

### Ethical considerations

No patient identifiable data was used in the analysis described here. The original QICKD study was approved by the Oxford Research Ethics Committee (Committee C). The ethical considerations of the QICKD study are described elsewhere [19,34].

## Results

A total of 741,913 people were included for analysis, of whom 35.914 (4.8%) had diabetes. We excluded people who died or left the practice before the start of the follow up period (n = 123), or if they were aged less than 18 at the start of the follow up period (n = 142,533). From this remaining adult cohort 35,502 people met the diagnostic criteria for diabetes.

The mean age of this cohort was 63.6 years (standard deviation of 14.3 years). Males made up 54% of the population. Diabetes was coded as type 1 diabetes in 1,202 (3.4%) people and type 2 in 30,767 (86.7%). The remaining 3,533 (9.9%) had other codes consistent with a diagnosis of diabetes or investigations consistent with the diagnostic criteria but where diabetes type was not specified.

**Figure 2:**
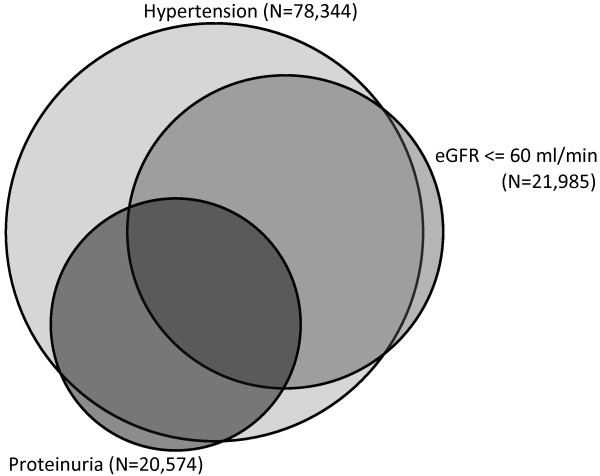
Area proportional Venn diagram demonstrating the interrelationship between hypertension, reduced eGFR, and proteinuria in people with diabetes.

Overall 15,813 (44.5%) people were identified with CKD. Hypertension was found to be present in almost everyone with diabetes and CKD; 15,244 (96.4%) (Figure 2). 5,862 people were found to have hypertension without CKD.

**Figure 3:**
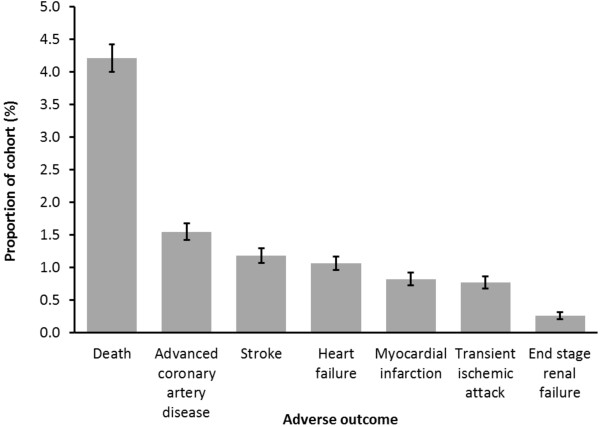
Adverse outcomes during 30 month follow up of 35,502 people with diabetes.

A total of 3,144 people (8.9%) in the cohort had an adverse outcome during the 30 month follow up period (Figure 3). All-cause mortality was the most common adverse outcome with 1,495 cases (4.2%), equating to an annual mortality of 17 per 1,000 person years. Non-fatal cardiovascular events occurred in a total of 1,531 people (4.3%). 1,691 (4.8%) people were lost to follow up before suffering an adverse event. These people were included in our final analysis. The demographics of the population are provided in Table 1.

**Table 1:** Clinical characteristics of the 35,502 people with diabetes included for analysis

**Characteristic**	**n (%)**	**Characteristic**	**n (%)**
Age: mean (±SD)	63.6 years (±14.3)	**Investigations**	
Gender: female	16,322 (45.97)	ACR: not monitored	13,104 (36.91)
Gender: male	19,180 (54.03)	ACR: normal	16,960 (56.00)
**Ethnicity**		ACR: microalbuminuria	4,623 (13.02)
White	15,222 (42.9)	ACR: macroalbuminuria	815 (2.30)
Mixed	406 (1.1)	HbA1c: not measured	3,435 (9.7)
Asian	6,215 (17.5)	HbA1c: < 7.5%	19,648 (55.3)
Black	2,183 (6.1)	HbA1c: > 7.5%	12,419 (35.0)
Other	2,187 (6.1)	Total cholesterol: not monitored	2,046 (5.8)
Not recorded	9,289 (26.2)	Total cholesterol: < 5.0 mmol/l	31,553 (88.9)
**Smoking Status**		Total cholesterol: > 5.0 mmol/l	1,903 (5.4)
Not recorded	7,474 (21.05)	LDL cholesterol: not monitored	15,678 (44.16)
Never smoked	16,349 (46.10)	LDL cholesterol: normal	14,612 (41.16)
Current Smoker	6,620 (18.65)	LDL cholesterol: high	5,212 (14.68)
Ex-smoker	5,059 (14.25)	HDL cholesterol: not monitored	5,850 (16.48)
**Alcohol Consumption**		HDL cholesterol: normal	24,372 (68.65)
Not recorded	12,272 (34.57)	HDL cholesterol: low	5,280 (14.87)
No alcohol	11,250 (31.70)	**No Hypertension**	
Light alcohol	10,124 (28.52)	Renal function not monitored	2,200 (6.20)
Excess alcohol	1,725 (4.86)	No CKD	15,229 (42.90)
Previous excess alcohol	131 (0.37)	CKD all stages	
**Comorbidities**		**Hypertension**	569 (1.60)
Heart failure	1,550 (4.37)	Renal function not monitored	2,260 (6.37)
Dialysis	97 (0.27)	No CKD	5,862 (16.51)
Stroke	1,438 (4.05)	CKD stages 1–2	7,581 (21.35)
Transient ischaemic attack	1,060 (2.99)	CKD stages 3–5	3,987 (11.23)
Ischaemic heart disease	6,328 (17.82)	CKD stages 3–5 with proteinuria	3,676 (10.35)
Coronary artery operation	2,322 (6.54)	LDL cholesterol: not monitored	15,678 (44.16)
Myocardial infarction	1,895 (5.34)		

Abbreviation: *SD*, Standard deviation.

A diagnosis of heart failure, stroke, TIA, ischaemic heart disease, or MI before the start of follow up were all found to be independent predictors of adverse outcome (Table 2). No association with adverse outcome was found with ethnicity, BMI, HbA1c, total cholesterol, and the use of aspirin. These variables were therefore removed from the multilevel logistic regression model.

**Table 2:** Univariate and multivariate odds ratios of an adverse outcome over 30 months of follow up, adjusted for CKD and hypertension

	**Univariate analysis**		**Multivariate analysis (MLM)**	
**Characteristic:**	**Odds ratio (95% CI)**	**P =**	**Odds ratio (95% CI)**	**P =**
Age (years)	1.06 (1.06-1.06)	<0.001	1.05 (1.05-1.05)	<0.001
Gender: female	1.00 (reference)	-	1.00 (reference)	-
Gender: male	1.21 (1.13-1.31)	<0.001	1.24 (1.14-1.36)	<0.001
Ethnicity				
White	1.00 (reference)	-	-	-
Mixed	0.61 (0.41-0.92)	0.019	-	-
Asian	1.00 (0.99-1.01)	0.488	-	-
Black	0.98 (0.97-1.00)	0.016	-	-
Other	0.95 (0.80-1.13)	0.591	-	-
Missing	1.02 (0.94-1.11)	0.640	-	-
Smoking Status				
Not recorded	1.18 (1.07-1.31)	0.002	0.95 (0.84-1.08)	0.472
Never smoked	1.00 (reference)	-	1.00 (reference)	-
Current Smoker	1.39 (1.24-1.57)	<0.001	1.38 (1.24-1.54)	<0.001
Ex-smoker	1.82 (1.61-2.06)	<0.001	1.18 (1.05-1.32)	0.005
Alcohol Consumption				
Not recorded	0.93 (0.85-1.01)	0.090	1.07 (0.96-1.20)	0.223
No alcohol	1.00 (reference)	-	1.00 (reference)	-
Light alcohol	0.87 (0.79-0.96)	0.004	0.88 (0.79-0.97)	0.013
Excess alcohol	1.04 (0.87-1.23)	0.688	1.24 (1.03-1.49)	0.024
Previous excess alcohol	1.16 (0.66-2.02)	0.610	1.04 (0.56-1.93)	0.895
Comorbidities				
BMI (kg/m^2^)	1.01 (0.99-1.02)	0.276	-	-
Heart failure	3.39 (2.99-3.83)	<0.001	1.30 (1.13-1.49)	<0.001
Dialysis	3.03 (1.88-4.89)	<0.001	1.76 (1.04-2.99)	0.036
Stroke	3.96 (3.49-4.48)	<0.001	2.26 (1.98-2.59)	<0.001
Transient ischaemic attack	3.27 (2.82-3.79)	<0.001	1.58 (1.34-1.86)	<0.001
Ischaemic heart disease	3.03 (2.80-3.27)	<0.001	1.64 (1.47-1.84)	<0.001
Coronary artery operation	2.63 (2.35-2.94)	<0.001	1.26 (1.09-1.44)	0.001
Myocardial infarction	2.88 (2.56-3.24)	<0.001	1.23 (1.06-1.42)	0.005
Investigations				
ACR: not monitored	0.77 (0.71-0.84)	<0.001	1.33 (1.19-1.50)	<0.001
ACR: normal	1.00 (reference)	-	1.00 (reference)	-
ACR: microalbuminuria	1.46 (1.31-1.62)	<0.001	1.16 (1.01-1.32)	0.034
ACR: macroalbuminuria	2.70 (2.26-3.23)	<0.001	2.32 (1.88-2.86)	<0.001
HbA1c: not measured	0.99 (0.98-1.01)	0.226	-	-
HbA1c: < 7.5%	1.00 (reference)	-	-	-
HbA1c: > 7.5%	1.16 (1.01-1.33)	0.224	-	-
Total cholesterol: not monitored	0.89 (0.76-1.05)	0.160	-	-
Total cholesterol: < 5.0 mmol/l	1.00 (reference)	-	-	-
Total cholesterol: > 5.0 mmol/l	0.88 (0.74-1.04)	0.134	-	-
LDL cholesterol: not monitored	1.13 (1.05-1.22)	0.002	1.23 (1.08-1.40)	0.002
LDL cholesterol: normal	1.00 (reference)	-	1.00 (reference)	-
LDL cholesterol: high	0.85 (0.75-0.96)	0.007	1.15 (1.01-1.31)	0.030
HDL cholesterol: not monitored	1.09 (0.98-1.20)	0.106	1.19 (1.02-1.39)	0.025
HDL cholesterol: normal	1.00 (reference)	-	1.00 (reference)	-
HDL cholesterol: low	1.13 (1.02-1.25)	0.022	1.17 (1.05-1.31)	0.006
**Multilevel model performance:**			**Random effects:**	
Bayesian information criteria (BIC)	19022		Intercepts for GP practice:	
Log-likelihood	-9312		Variance	0.111
ROC curve statistic	0.767		Standard deviation	0.334

Abbreviations: *MLM*, multilevel model; *CI*, confidence interval.

**Figure 4:**
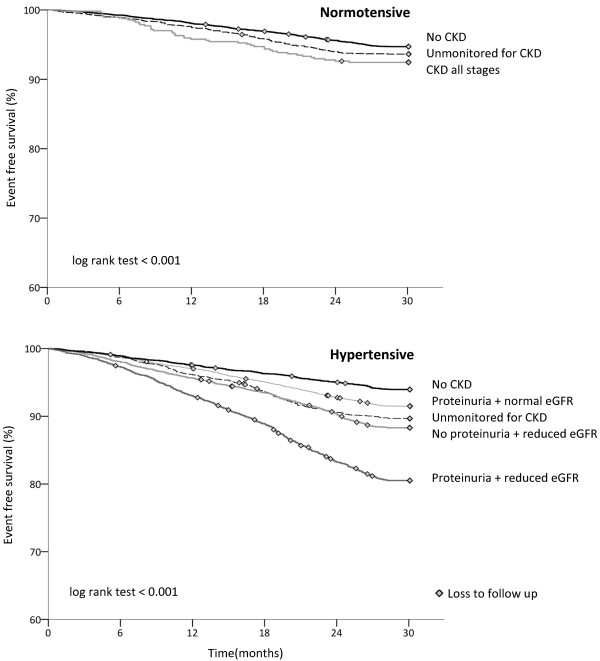
Kaplan-Meier event free survival curves by CKD category in people with normotension and hypertension.

The odd ratios for adverse outcomes increased with declining renal function, proteinuria, and hypertension (Table 3). Cox regression analysis also demonstrates increasing hazard ratios with declining renal function (Table 4). Event free survival analysis for people with CKD with and without hypertension showed their summative effect on adverse outcomes (Figure 4).

**Table 3:** Adjusted odd ratios for vascular and renal events and mortality of 35,502 people with diabetes, over 30 months of follow up, by CKD and hypertension category

	**Univariate analysis**		**Multivariate analysis (MLM)**	
	**Odds ratio (95% CI)**	**P =**	**Odds ratio (95% CI)**	**P =**
No Hypertension				
Renal function not monitored	1.21 (1.00-1.47)	0.053	1.32 (1.07-1.64)	0.011
No CKD	1.00 (reference)		1.00 (reference)	-
CKD all stages	3.86 (2.15-6.94)	<0.001	1.34 (0.93-1.92)	0.117
Hypertension				
Renal function not monitored	2.07 (1.76-2.43)	<0.001	1.35 (1.13-1.63)	0.001
No CKD	1.16 (1.01-1.33)	0.038	0.97 (0.84-1.11)	0.629
CKD stages 1–2	1.66 (1.48-1.88)	<0.001	1.19 (1.03-1.36)	0.015
CKD stages 3–5	2.39 (2.10-2.73)	<0.001	1.13 (0.98-1.31)	0.090
CKD stages 3–5 with proteinuria	4.38 (3.88-4.94)	<0.001	1.58 (1.36-1.83)	<0.001

Abbreviation: *CI*, confidence interval.

**Table 4:** Adjusted hazard ratios (Cox regression analysis) for adverse outcomes in 35,502 people with diabetes, over 30 months of follow up, by CKD and hypertension category

	**Hazard ratio (95% CI)**	**P =**
No Hypertension		
Renal function not monitored	1.49 (1.23-1.80)	0.011
No CKD	1.00 (reference)	-
CKD all stages	1.62 (0.76-3.42)	0.060
Hypertension		
Renal function not monitored	1.55 (1.32-1.82)	<0.001
No CKD	0.98 (0.85-1.12)	0.765
CKD stages 1–2	1.31 (1.16-1.47)	<0.001
CKD stages 3–5	1.14 (1.00-1.31)	0.050
CKD stages 3–5 with proteinuria	1.72 (1.52-1.95)	<0.001

Abbreviation: *CI*, confidence interval.

A total of 4,460 (12.6%) people had incomplete CKD screening during the 2.5 year baseline period. This comprised 1,574 (4.4%) people with no serum creatinine recorded and 3,478 (9.8%) untested for proteinuria. People with unmonitored renal function, both with and without hypertension, were found to have significantly higher incidence of adverse vascular and renal outcomes than those with normal renal function. People with unmonitored renal function were found to have lower prescription rates of ACE inhibitors and ARBs (41.4%, 95% CI 40.2-42.6%) than people with no evidence of CKD (54.8%, 95% CI 54.1-55.4%) and people with CKD (71.1%, 95% CI 70.3-71.9%). They were also younger, more likely to drink excessive alcohol and smoke, had lower prescription rates of other medications, were more likely to have other missing data, and had worse mean cholesterol values (Table 5).

**Table 5:** Characteristics of people without monitored renal function compared to those with monitored renal function

	**Renal function not monitored (N = 4460) n (%)**	**Renal function monitored (N = 31042) n (%)**	**P =**
Age: mean (±SD)	59.1 years (±16.5)	64.2 years (±13.9)	<0.001
Gender: male	2,434 (54.6%)	16,764 (53.9%)	0.432
Ethnicity	N = 2,227	N = 23,986	
White	1,232 (55.3)	13,990 (58.3)	
Mixed	37 (1.7)	369 (1.5)	
Asian	480 (21.6)	5,735 (23.9)	
Black	210 (9.4)	1,973 (8.2)	
Other	268 (12.0)	1,919 (8.0)	<0.001
Smoking Status	N = 2765	N = 25263	
Never smoked	1,562 (56.5)	14,787 (58.5)	
Current Smoker	741 (26.8)	5,879 (18.2)	
Ex-smoker	462 (16.7)	4,597 (18.2)	<0.001
Alcohol Consumption	N = 1,662	N = 21568	
No alcohol	736 (44.3)	10,514 (48.7)	
Light alcohol	707 (42.5)	9,417 (43.7)	
Excess alcohol	194 (11.7)	1,531 (7.1)	
Previous excess alcohol	25 (1.5)	106 (0.5)	<0.001
Investigations			
HbA1c: not monitored	1,496 (33.5)	1,939 (6.2)	<0.001
HbA1c: mean (±SD)	8.16% (±2.09)	8.55 (±2.07)	<0.001
Total cholesterol: not monitored	1,413 (31.7)	633 (2.0)	<0.001
Total cholesterol: mean (±SD)	4.93 mmol/l (±1.15)	4.60 mmol/l (±1.08)	<0.001
LDL cholesterol: not monitored	2,510 (56.3)	13,168 (42.4)	<0.001
LDL cholesterol: mean (±SD)	2.70 mmol/l (±0.99)	2.51 mmol/l (±0.91)	<0.001
HDL cholesterol: not monitored	1,688 (37.8)	4,162 (13.4)	<0.001
HDL cholesterol: mean (±SD)	1.35 mmol/l (±0.39)	1.29 mmol/l (±0.38)	<0.001
Medications			
ACE/ARBs prescribed	1,847 (41.4)	20,403 (65.7)	<0.001
Lipid lowering drugs prescribed	2,134 (47.8)	24,136 (77.8)	<0.001
Aspirin prescribed	801 (18.0)	9,081 (29.3)	<0.001

For ethnicity, smoking status, and alcohol consumption only those with recorded values are included. Abbreviations: *SD*, standard deviation.

## Discussion

### Principal findings

We report three principal findings: Both reduced eGFR, and proteinuria are independent predictors of adverse outcomes. Nearly everyone with diabetes and CKD has hypertension, using the diagnostic threshold recommended by national guidelines. People with unmonitored renal function do worse that those who are monitored and appear to receive suboptimal medical therapies.

The combination of proteinuria and reduced eGFR is associated with the highest risk of adverse outcomes in people with and without hypertension. In people with hypertension and normal eGFR, proteinuria, also significantly increases the odds of adverse outcomes.

Almost all patients with CKD had hypertension whether their CKD was diagnosed by the presence of proteinuria or reduced eGFR. There were, however, a considerable number of people with hypertension and no evidence of renal impairment. However, CKD testing in people with diabetes and hypertension is currently suboptimal: just over 10% of people had not had appropriate monitoring for CKD by measurement of eGFR and assessment for proteinuria.

People with unmonitored renal function have a higher risk of adverse outcomes than those with normal renal function. This group has the lowest prescription rates of antihypertensive medication, lipid lowering drugs, and aspirin, suggesting people in this group receive suboptimal therapy. They also had higher rates of excess alcohol use and smoking. Whether these differences reflect variation in provision of healthcare or in attitudes to healthcare remains uncertain.

### Implications of the findings

This study highlights the importance of frequent monitoring of eGFR and proteinuria in people with diabetes. In particular, careful monitoring of renal function and testing for proteinuria is essential for people with hypertension and diabetes due to the very high proportion of people with renal impairment in this group.

It is plausible that failure to monitor renal function is associated with wider neglect in clinical management although whether this is due to patient factors or healthcare factors is unclear. Computerised prompts and other recall systems should perhaps focus on those at highest risk i.e. not monitored and not prescribed antihypertensive medication.

### Comparison with literature

The prevalence of CKD stages 3–5 in people with hypertension and diabetes is reported to be 43%, [35] in close agreement with our value 36.3%; and other community studies report a similar association between CKD and hypertension in people with diabetes [36]. However, we find this proportion increases to 72.2% if people with CKD stage 1 and 2 are included.

Previous studies in the UK report the prevalence of eGFR screening to be 92–82% in people with diabetes over a 2 year period [11,37] compared with 95.6% over 30 months in our study. The prevalence of ACR screening is reported as 55.2% over 2 years [37], again very similar to our study (63.1%). The association between absence of assessment for CKD and vascular and renal outcomes has not previously been investigated; though we have previously reported that people not on diabetes disease registers appear to receive suboptimal care [38].

A similar additive association between the proteinuria and eGFR components of renal disease has been reported with vascular and renal events and mortality in the general population and, high risk groups and in people with diabetes [7-9,39-41]. However the combined impact of these factors with hypertension has not previously been explored in diabetes.

### Limitations of the method

The limitations of this study include those of using routine data [42]. In particular the potential for ascertainment bias in data categories where a significant proportion of data has not been collected (e.g. smoking status and alcohol consumption). For this reason we have included the complete population for analysis with ‘;not recorded’ categories incorporated to identify potential associations with unrecorded data and adverse outcomes. Smoking status and alcohol consumption not recorded were not significantly associated with worse outcomes; OR 0.95 (95% CI 0.84-1.08) and OR 1.07 (95% CI 0.96-1.20) respectively.

Urine samples are frequently tested by clinicians using reagent strips, the results of which may not always be coded, resulting in underreporting of proteinuria testing. However, it is likely that there is a bias for coding positive test results which would not explain the increased risk of adverse outcomes in the apparently unmonitored group.

The causative factors for increased adverse outcomes in people with unmonitored renal function cannot be fully determined. Therefore the benefit of improving screening in this unmonitored group cannot be directly established.

Even with a large cohort the short duration of follow-up (2.5 years) is also a limitation here. A longer follow-up period, ideally at least five years would be preferable. Many things, including assessing health economic impact would be possible to estimate given change over 5 years or more. The large sample size makes possible lack of power to identify significant associations unlikely in this study, although weak associations may still be missed.

We have previously demonstrated that the population demographics of the QICKD cohort provide a close age and gender match to the diversity of the English population (from census data) although ethnic minorities appear to be under represented [43]. These results should not, however, be extrapolated to dissimilar populations or different healthcare settings.

### Further research

Further work is needed to demonstrate whether improved screening of renal function in people with diabetes leads in a reduction in vascular and renal events and mortality.

## Conclusions

CKD and proteinuria are associated with worse health outcomes in people with diabetes, and this effect is additive. People with diabetes, who are not monitored for renal disease, have an increased risk of adverse events and appear to receive suboptimal medical therapy.

## Competing interests

The authors declare no competing interests.

## Authors’ contributions

APM, BR, SJ, JNvV, and HL performed the statistical analysis. JNvV, and HL performed data processing and database handling. APM, BR, and SdeL wrote the manuscript. HG, CRVT, KK, and KH provided review of the manuscript and contributed to the final write-up. SdeL was the senior study investigator. All authors read and approved the final manuscript.

## Pre-publication history

The pre-publication history for this paper can be accessed here:

http://www.biomedcentral.com/1471-2369/14/198/prepub
